# Pleiotropic Effects of Sodium-Glucose Cotransporter-2 Inhibitors in Cardiovascular Disease and Chronic Kidney Disease

**DOI:** 10.3390/jcm12082824

**Published:** 2023-04-12

**Authors:** Anjay Rastogi, James L. Januzzi

**Affiliations:** 1 David Geffen School of Medicine, University of California, Los Angeles, CA 90095, USA; 2 Massachusetts General Hospital, Boston, MA 02114, USA; jjanuzzi@partners.org; 3 Department of Medicine, Harvard Medical School, Boston, MA 02115, USA; 4 Baim Institute for Clinical Research, Boston, MA 02215, USA

**Keywords:** cardiac remodeling, cardiovascular outcomes, chronic kidney disease, glomerular pressure, heart failure, mechanism of action, renal outcomes, sodium-glucose cotransporter-2 inhibitors, sympathetic nervous system, type 2 diabetes

## Abstract

Sodium-glucose cotransporter-2 inhibitors (SGLT2is) have been shown to improve cardiovascular and renal outcomes in patients with established cardiovascular disease, chronic kidney disease (CKD), and heart failure (HF) with reduced or preserved ejection fraction. Clinical benefit has been substantiated in patients with and without type 2 diabetes (T2D). Consequently, SGLT2is have an increasingly important role in HF and CKD management that extends beyond T2D treatment. Their pleiotropic pharmacological effects underlying their cardiovascular and renal benefits are not completely understood but include significant effects beyond blood glucose reduction. SGLT2is inhibit the reabsorption of glucose and sodium in the proximal tubule which, in addition to lowering blood glucose, activates tubuloglomerular feedback, leading to reduced glomerular hydrostatic pressure and the mitigation of glomerular filtration rate loss. SGLT2is have diuretic and natriuretic effects, leading to decreased blood pressure, preload, and left ventricular (LV) filling pressure, and improvements in other surrogates of afterload. In HF, SGLT2is mitigate the risks of hyperkalemia and ventricular arrhythmia and improve LV dysfunction. SGLT2is also reduce sympathetic tone and uric acid levels, increase hemoglobin levels, and are postulated to have anti-inflammatory properties. This narrative review discusses the multifactorial and interrelated pharmacological mechanisms underlying the cardiovascular and renal benefits of SGLT2is.

## 1. Introduction

Cardiovascular disease (CVD), chronic kidney disease (CKD), and type 2 diabetes (T2D) are chronic progressive diseases with considerable overlap. Almost half (49%) of US adults aged ≥20 years have CVD (defined as coronary heart disease, heart failure [HF], stroke, and hypertension); the prevalence of CKD and T2D among US adults is approximately 15% and 10%, respectively [1].

Pathologies of the cardiovascular and renal systems are linked, with disorders of one system frequently involving the other in a cardiorenal continuum [2]. Patients with T2D have increased risk of developing macrovascular (e.g., myocardial infarction, stroke, and peripheral artery disease) and microvascular (i.e., nephropathy, retinopathy, and neuropathy) complications that cause significant morbidity and mortality [3,4,5]. T2D is an independent risk factor for HF, and the incidence of HF is approximately fourfold higher in patients with T2D compared with that in the general population; furthermore, outcomes are worse when these conditions occur in the same patient [6]. T2D is the most common cause of CKD [7], and CKD is associated with an increased risk of cardiovascular events [8,9]. 

In the last decade, sodium-glucose cotransporter-2 inhibitors (SGLT2is) have been shown to provide clinical benefits across the cardiorenal spectrum. SGLT2is were originally developed and approved as glucose-lowering drugs for patients with T2D [10,11,12,13]. Subsequently, cardiovascular safety trials mandated by the US Food and Drug Administration found that SGLT2i treatment was associated with improved cardiovascular outcomes in patients with T2D [14,15,16]. These cardiovascular outcomes trials (CVOTs) and additional renal outcomes studies found that SGLT2is reduced the risk of major adverse cardiovascular events (MACEs) and renal events in patients with T2D and established atherosclerotic CVD, multiple cardiovascular risk factors, or CKD (Table 1) [14,15,17,18,19,20,21]. More recently, SGLT2is have been shown to confer benefit in patients with HF across the full spectrum of ejection fractions (EFs) [22,23,24,25,26] and in patients with CKD, regardless of their glycemic status (Table 1) [27,28,29]. On the basis of the findings from these outcome studies, drugs in this class have been approved for use in patients with established CVD or multiple risk factors [10,11], CKD [10,12], HF with reduced EF (HFrEF) [10,11], and HF with preserved EF (HFpEF) [11], including in patients without T2D [10,12], for the purpose of reducing the risk of cardiovascular death or hospitalization and preventing disease progression.

US and European treatment guidelines recommend SGLT2is to reduce the risk of HF, MACE, cardiovascular death, and progression of kidney disease in patients with T2D with established CVD or multiple risk factors for CVD [31,32,33,34,35,36] and for the treatment of HF and CKD in patients with or without T2D [34,35,36]. Although the first SGLT2i approvals for HF were for HFrEF, the updated 2022 American Heart Association (AHA)/American College of Cardiology (ACC)/Heart Failure Society of America (HFSA) guideline for the management of HF recommends the use of an SGLT2i across the full spectrum of HF, including HFrEF (defined as left ventricular EF [LVEF] ≤ 40%), HF with mildly reduced EF (LVEF 41–49%), and HFpEF (LVEF ≥50%), irrespective of the presence of T2D [36]. 

SGLT2is have pleiotropic pharmacological effects that include those related to their glucose-lowering effects and those that are independent of glucose lowering effects. Some pharmacological mechanisms, such as the effects on sodium-glucose cotransporter-2 (SGLT2), are well understood and linked to evidence in clinical outcome studies. Other mechanisms are putative, based on small clinical studies or preclinical data, and their relationship to clinical outcomes is less certain. In particular, the mechanistic links between SGLT2is and the clinical benefits observed in large CVOTs have not been fully elucidated. The aim of this narrative review is to provide an overview of the glucose-independent mechanisms that may contribute to the cardiovascular and renal protective effects of SGLT2is irrespective of a patient’s glycemic status.

## 2. Materials and Methods

A targeted literature search of PubMed was conducted to identify relevant English-language publications using the following search terms: (“SGLT2 inhibitors”, “SGLT-2i”, “SGLT2i”, “sodium-glucose cotransporter 2 inhibitors”, “sodium-glucose cotransporter-2 inhibitors”, “canagliflozin”, “dapagliflozin”, “empagliflozin”, “ertugliflozin”, “empagliflozin”, “sotagliflozin”) AND (“heart failure”, “chronic kidney disease”, “type 2 diabetes mellitus”, “non-insulin dependent diabetes”) AND (“mechanism of action”, “glomerular pressure”, “sympathetic nervous system”, “hematocrit”, “uric acid”, “electrolytes”, “preload/afterload”). English-language papers published between July 2015 and December 2022 reporting clinical studies and meta-analyses of SGLT2is were identified. Additional relevant studies were identified from the bibliographies of these publications. 

## 3. SGLT2i Mechanism of Action

### 3.1. Inhibition of SGLT2 and Glucose-Lowering Effects

Under normal physiological conditions, the kidneys contribute to glucose homeostasis by reabsorbing almost all of the glucose in the glomerular filtrate with a negligible amount excreted in urine [37]. This is achieved primarily by SGLT2 (encoded by *SLC5A2*), which is expressed almost exclusively in the kidney, whereas sodium-glucose cotransporter-1 (*SLC5A1*) is expressed mainly in the small intestine and to a small extent in the kidney [38]. SGLT2 is a membrane transport protein located exclusively in the apical membrane of epithelial cells in the proximal tubule that facilitates reabsorption of approximately 90% of filtered glucose coupled with sodium transport in a 1:1 ratio. This passive process involves an electrochemical gradient—sodium ions flow from a region of high concentration in the glomerular filtrate to a region of low concentration in tubular epithelial cells—maintained by the basolateral Na+/K+ ATPase pump [39]. After being reabsorbed from the glomerular filtrate, glucose is transported from the tubular epithelial cells to the interstitial fluid via glucose transporter 2, the main glucose transporter in the kidney, which is located in the basolateral membrane [40,41].

Inhibition of SGLT2 blocks reabsorption and increases renal excretion of glucose [42]. This results in reductions in plasma glucose levels in patients with T2D that are reflected by reductions in glycated hemoglobin (A1C) in the order of ~1% [43,44,45,46]. This effect is markedly reduced as the glomerular filtration rate (GFR) decreases to 45 mL/min/1.73 m^2^ and is negligible in patients with a GFR of 30 mL/min/1.73 m^2^ or lower [47,48,49]. Inhibition of renal glucose reabsorption and reductions in plasma glucose levels achieved with SGLT2is occur independent of insulin levels or peripheral insulin resistance; thus, the risk of hypoglycemia is low with SGLT2i therapy [5,50]. By promoting glucosuria, treatment with an SGLT2i is associated with a daily caloric loss of approximately 200 kcal/day and weight loss of approximately 2–3 kg over 6 months [51,52].

### 3.2. Reduction in Intraglomerular Pressure and Preservation of Renal Function

**Figure 1:**
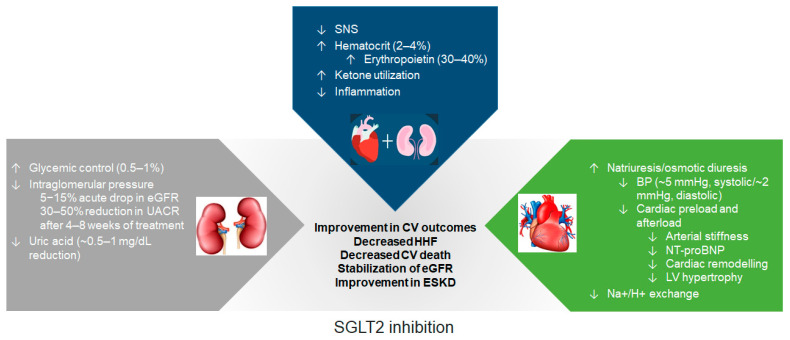
Mechanisms underlying the cardio- and renoprotective effects of SGLT2is. BP, blood pressure; CV, cardiovascular; eGFR, estimated glomerular filtration rate; ESKD, end-stage kidney disease; H^+^, hydrogen; HHF, hospitalization for heart failure; LV, left ventricular; Na^+^, sodium; NT-proBNP, N-terminal prohormone of brain natriuretic peptide; SGLT2, sodium-glucose cotransporter-2; SGLT2i, sodium-glucose cotransporter-2 inhibitor; SNS, sympathetic nervous system; UACR, urinary albumin-to-creatinine ratio.

The cardiorenal effects of SGLT2is are not the result of glucose-lowering activity alone, because they are observed sooner than would be expected for a glucose-mediated mechanism of action, are sustained with continued use, and are independent of T2D status. Moreover, cardiorenal benefits are not observed with most classes of anti-hyperglycemic drugs [53]. This review focuses on the mechanisms for which clinical data are available (Figure 1).

Glomerular filtration is regulated, in part, by tubuloglomerular feedback. Increasing sodium chloride concentrations in the distal tubule cause a release of adenosine from cells in the macula densa, which results in constriction of afferent (precapillary) glomerular arterioles and a reduction in the GFR. The expression and activity of SGLT2 are increased in patients with T2D [54], resulting in increased renal reabsorption of glucose and sodium in these individuals. Increased reabsorption of sodium in the proximal tubule reduces the concentration of sodium chloride in the macula densa and leads to the deactivation of tubuloglomerular feedback and dilatation of afferent arterioles [55,56]. Concurrent activation of the renin–angiotensin–aldosterone system (RAAS) induces constriction of efferent (postcapillary) glomerular arterioles, and together these changes cause persistent increases in single-nephron GFR, glomerular hyperfiltration, and glomerular hypertension [56]. Glomerular hyperfiltration is a common cause of kidney injury and leads to progressive reduction of kidney function [57].

**Figure 2:**
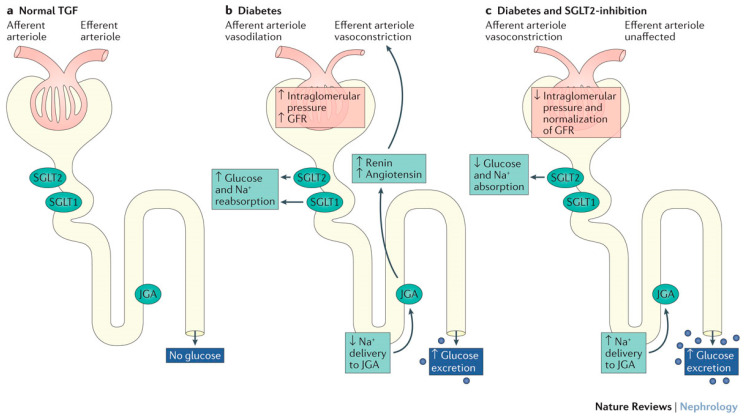
Glomerular effects of SGLT2is. In states of normal TGF, no glucose is delivered to the JGA. However, with glucosuria, TGF is abnormal, resulting in increased efferent arteriolar constriction and hyperfiltration. With SGLT2 inhibition, restoration of TGF occurs, lowering intraglomerular pressure and normalization of GFR. Reprintede with permission from DeFronzo RA, et al. *Nat Rev Nephrol.* 2017 [58]. Copyright © 2016, Nature Publishing Group, a division of Macmillan Publishers Ltd. All rights reserved. GFR, glomerular filtration rate; JGA, juxtaglomerular apparatus; Na^+^, sodium; SGLT1, sodium-glucose cotransporter 1; SGLT2, sodium-glucose cotransporter 2; SGLT2i, sodium-glucose cotransporter 2 inhibitor; TGF, tubular glomerular feedback.

SGLT2i therapy mitigates these effects by blocking the reabsorption of glucose and sodium in the proximal tubule. Solute delivery to the macula densa is restored and tubuloglomerular feedback is activated, which results in constriction of afferent arterioles, reduced intraglomerular pressure, and decreased hyperfiltration (Figure 2) [37,57,58,59]. Reduced glomerular hydrostatic pressure preserves renal function in the long term [59].

Clinically, the restoration of tubuloglomerular feedback manifests as an acute decrease in the estimated glomerular filtration rate (eGFR) of ~5 mL/min/1.73 m^2^ during the first 2–6 weeks of SGLT2i treatment [30,60,61,62,63,64,65,66,67]. After this initial decline, the eGFR recovers and returns to baseline, indicating that the initial drop is the result of altered hemodynamics rather than glomerular damage [53,62,63,64]. This initial eGFR decrease was not found to be associated with greater long-term decreases in eGFR [66,67]. SGLT2is confer renoprotective effects irrespective of baseline eGFR and CKD stage [30,68], and long-term SGLT2i therapy preserves kidney function in patients with and without T2D [60,69]. In DECLARE-TIMI 58, treatment with dapagliflozin significantly reduced the progression of kidney disease relative to a placebo in patients with T2D. Over a median of 4.2 years of follow-up, significantly fewer dapagliflozin-treated versus placebo-treated patients experienced a sustained decrease in eGFR, defined as a 40% decrease from baseline to <60 mL/min/1.73 m^2^ or progression to end-stage kidney disease [69]. Therefore, a transient decrease in eGFR after treatment initiation should not prompt interruption or discontinuation of SGLT2i therapy [34]. Rather, the data suggest that treatment with an SGLT2i prevents CKD in patients with T2D.

Data from EMPA-REG OUTCOME [14,63], DECLARE-TIMI 58 [18,69], DELIVER [25,30], VERTIS-CV [19,62], and CREDENCE [28,64] suggest that the early eGFR decrease should not be cause for concern unless there are signs and symptoms of volume depletion (orthostatic hypotension, blood pressure [BP] <120/70 mmHg), specifically in older patients (>65–70 years) or in those receiving high-dose diuretics [70].

SGLT2i treatment significantly reduces albumin excretion in patients with T2D and microalbuminuria (urinary albumin-to-creatinine ratio [UACR] of 30–300 mg/g) or macroalbuminuria (UACR >300 mg/g), including patients receiving drugs that block the RAAS [71,72,73]. A prespecified analysis of the DAPA-CKD study showed that significant reductions in UACR occurred in patients with and without T2D (with larger reductions in patients with T2D) [74]. Similarly, an exploratory analysis of DECLARE-TIMI 58 showed that dapagliflozin therapy slowed the increase in UACR over 4 years, regardless of baseline eGFR and UACR, including in non-albuminuric patients [75]. The positive effect on albumin excretion is reversed if SGLT2i therapy is discontinued, which indicates that it is the result of altered renal hemodynamics [71,72,73]. SGLT2is have also been shown to slow the long-term decline in eGFR in patients with T2D and microalbuminuria or macroalbuminuria [76]. 

Acute kidney injury (AKI) has been reported in the post-marketing surveillance of patients taking SGLT2is, although a causal relationship has not been established [10,11,12,13]. It is possible that SGLT2i-associated AKI is related to volume depletion, an uncommon adverse event in patients taking SGLT2is. Consistent with this hypothesis, the risk of AKI was low in patients receiving an SGLT2i in CVOTs [15,17,18,27,28]. Moreover, a meta-analysis of data from CVOTs showed a 34% reduction in the risk of AKI during SGLT2i therapy, relative to placebo [77]. Similarly, prespecified analyses of DAPA-CKD [78] and DAPA-HF [79] showed that dapagliflozin reduced the risk of abrupt declines in kidney function, defined as a doubling of serum creatinine by 32% [78] and 44% [79], relative to placebo.

### 3.3. Reduction in Preload/Afterload and Effects on BP

SGLT2i therapy has diuretic and natriuretic effects in patients with T2D. This results in an approximate 1–2 kg decrease in total body water, a 6% loss in sodium, and a decrease in plasma volume during the first few weeks of treatment [80,81,82]. However, these effects are transient and stabilize subsequently. The contraction in plasma volume decreases cardiac preload [59] and left ventricular (LV) filling pressure, leading to reductions in myocardial stretch and interstitial fibrosis [45]. 

Plasma volume contraction is associated with sustained reductions in systolic/diastolic BP (SBP/DBP) of approximately 5/2 mmHg without a compensatory increase in heart rate and no evidence of orthostatic hypotension [80,83,84,85]. These improvements in BP are amplified by the SGLT2i-associated reduction in body weight [80,83,84].

The diuretic effects of SGLT2is differ from those of thiazide or loop diuretics. SGLT2is do not affect plasma osmolality or reduce intravascular volume. Despite having less potent natriuretic and diuretic effects than a loop diuretic, dapagliflozin produced greater reductions in interstitial fluid volume than in blood volume [86]. This may explain why an SGLT2i may provide better control of congestion without reducing arterial filling and perfusion in patients with HF. From a clinical perspective, better control of congestion by SGLT2is may result in the lower hospitalization rates for HF observed in numerous CVOTs [14,15,18,19,21,28]. Importantly, unlike loop diuretics, the natriuretic effects of SGLT2i therapy do not result in activation of the RAAS [59]. Furthermore, reductions in BP did not affect natriuresis in studies with dapagliflozin and empagliflozin in patients with T2D and preserved kidney function [87,88]. The available data suggest the cardiovascular and renal protective effects of SGLT2is are mediated by factors other than natriuresis and volume changes and are independent of changes in mean SBP [45,53,87,88]. 

In patients with HFrEF, the natriuretic effects of SGLT2is are synergistic with loop diuretics and allow for reductions in the dose of loop diuretics [89,90]. For example, a significantly higher proportion of patients had their diuretic dose reduced after 6 months and 12 months of treatment with dapagliflozin in DAPA-HF [90].

SGLT2i treatment decreases sympathetic nervous system (SNS) activity (discussed later in this review), which is accompanied by reductions in central SBP, pulse pressure, mean arterial pressure (MAP), the double product (SBP multiplied by heart rate), and aortic pulse wave velocity, which are indications of reduced arterial stiffness and surrogates of afterload [91,92,93]. In addition, SGLT2i–mediated vasodilation via activation of voltage-gated potassium channels and protein kinase G may result in improved subendocardial blood flow [41,94]. A post hoc analysis of a large randomized controlled trial demonstrated favorable effects of empagliflozin on pulse pressure, MAP, and the double product after a median of 3.1 years of treatment [95].

### 3.4. Effects on Potassium Levels 

Hyperkalemia is common in patients with HF, is associated with specific risk factors including comorbidities such as CKD and T2D and drugs such as mineralocorticoid antagonists and RAAS inhibitors [96], and increases the risk of cardiovascular-related morbidity and mortality [97]. Treatment with an SGLT2i reduced the risks of moderate hyperkalemia in patients with T2D or CKD [98] and in patients with HFrEF and HFpEF, including patients receiving mineralocorticoid antagonists, in major CVOTs [99,100,101,102]. Importantly, SGLT2i use did not increase the incidence of hypokalemia in patients with T2D or CKD receiving loop or thiazide diuretics [90,102]. It is not clear whether dose modifications of mineralocorticoid antagonists, RAAS inhibitors, or diuretics contributed to the observed differences in hyperkalemia incidence. In a meta-analysis of data from six cardiovascular and renal outcome trials, the beneficial effects of SGLT2i therapy on the incidence of serious hyperkalemia were significantly different in patients who were and were not receiving RAAS inhibitors at baseline; however, treatment with an SGLT2i as compared with a placebo was associated with significantly lower risk of serious hyperkalemia in those who were and were not receiving RAAS inhibitors at baseline [98]. 

### 3.5. Effects on LV Size and Function

In patients with T2D and a history of CVD, SGLT2is reduce LV mass index and end-diastolic volume [45,103]. In patients with HFrEF with and without T2D, empagliflozin significantly reduced LV and atrial volumes [104,105,106]. In patients with HFpEF, dapagliflozin significantly improved LV diastolic functional parameters and global longitudinal strain [107]. Canagliflozin has also been shown to improve LV parameters in patients with HFpEF [108]. 

### 3.6. Risk of Ventricular Arrhythmias

Ventricular arrhythmias are common among patients with HFrEF, resulting from chamber dilatation and cardiomyocyte stretch, and may lead to sudden death. A post hoc analysis of the DAPA-HF study, which included patients with HFrEF both with and without T2D, showed that the addition of dapagliflozin to standard therapy for HF was associated with a 21% reduction in the risk of any ventricular arrhythmia, resuscitated cardiac arrest, or sudden death relative to the placebo plus standard therapy [109]. The reductions in cardiovascular outcomes were associated with reduced cardiac chamber size and decreased N-terminal prohormone of brain natriuretic peptide levels, which suggests that SGLT2i therapy decreases wall stress [109]. 

### 3.7. Reduction in SNS Activation

Chronic activation of the SNS is prevalent in patients with T2D, ischemic heart disease, HF, hypertension, and CKD and results in fluid retention, endothelial dysfunction, and increased arterial stiffness [110]; chronic SNS activation is associated with poor prognosis [111,112,113,114,115]. The SNS is regulated, in part, by the kidneys, as renal stress results in increased signaling to the brain, stimulating the sympathetic center and enhancing sympathetic outflow [110]. It has been proposed that transient osmotic diuresis induced by SGLT2is leads to a decrease in sympathetic outflow to the kidneys and results in a correction of systemic fluid retention in patients with T2D [110].

There is evidence that SGLT2is inhibit SNS activation by suppressing renal afferent signaling to the brain and central reflex mechanisms [110]. Dapagliflozin inhibited SNS activity in a neurogenic hypertensive mouse model as indicated by reduced tyrosine hydroxylase staining in renal tissue and norepinephrine levels in the kidney [116], and empagliflozin reduced renal sympathetic nerve overactivity in a rabbit model of diabetes [117]. Luseogliflozin reduced resting heart rate in patients with T2D and a baseline heart rate >70 beats per minute [118]. Empagliflozin significantly reduced the low:high frequency ratio in heart rate variability in patients with T2D and acute myocardial infarction [119]. Dapagliflozin significantly decreased muscle sympathetic nerve activity (MSNA) correlated with a decrease in brain natriuretic peptide in a small study in patients with T2D with and without HF [120]. The reduction in MSNA was more marked in the patients with HF, which suggests that the cardioprotective effects of SGLT2is are due in part to improved sympathetic nerve activity [120]. 

### 3.8. Effects on Hematologic Parameters 

Treatment with an SGLT2i is associated with improvements in hemoglobin, hematocrit, and erythropoietin levels. For example, in a short-term study in patients with T2D, serum erythropoietin levels and reticulocyte counts increased over the first 4 weeks of treatment with dapagliflozin, whereas increases in hematocrit, hemoglobin, and red cell mass were sustained over 12 weeks and were all significant when compared with those with a placebo or hydrochlorothiazide [80]. 

Significantly higher hematocrit and hemoglobin levels were observed over a median of 2.6 years of follow-up in patients with T2D and CKD in CREDENCE [121], over 206 weeks of follow-up in patients with T2D and CVD in EMPA-REG OUTCOME [122], over 260 weeks of follow-up in patients with T2D and CVD in VERTIS CV [123,124], and over 12 to 48 months of follow-up in patients with T2D and CVD in DECLARE-TIMI-58 [125]. Moreover, the risk of anemia, initiation of treatment with an iron preparation, or a requirement for an erythropoiesis-stimulating agent were all significantly lower in recipients of canagliflozin than in recipients of placebo in CREDENCE [121]. 

In patients with HFrEF enrolled in DAPA-HF, dapagliflozin treatment was associated with increases in hematocrit that plateaued after 4 months and were sustained for up to 2 years, regardless of T2D status [126], diuretic use, or baseline diuretic dose [90]. The increase in hematocrit persisted at 6 and 12 months regardless of whether the diuretic dose was increased, decreased, or remained unchanged [90].

The mechanism underlying these improvements has not been fully elucidated; however, given the weak diuretic effect of SGLT2is and the sustained nature of these hematologic changes, it seems likely that factors in addition to hemoconcentration are involved [80,90,122,125,127].

There is evidence that SGLT2is increase erythropoiesis by modulating iron regulatory proteins [128,129]. For example, in patients with T2D, dapagliflozin significantly reduced levels of hepcidin, which is a known suppressor of erythropoiesis, and increased levels of erythroferrone, which is a hepcidin inhibitor [128]. In addition, SGLT2is may improve the hypoxic microenvironment of the proximal tubule by alleviating metabolic stress, normalizing renal cortical oxygenation, and improving the tubulointerstitial function [127].

Improving hematologic status enhances the delivery of oxygen to tissues and may in part explain reductions in cardiovascular death in patients treated with SGLT2is [122,130,131].

### 3.9. Reduction in Uric Acid Levels

Hyperuricemia increases oxidative stress, impairs endothelial function, activates the RAAS, and is associated with an increased risk of hypertension, CVD, and CKD. Treatment with an SGLT2i is associated with sustained reductions in serum uric acid levels in patients with T2D [132,133,134] or HFrEF [135]. In patients with T2D, the magnitude of the reduction in serum uric acid levels decreased with increasing duration of disease, higher A1C levels, or decreasing eGFR [133]. Moreover, the uric acid lowering effect of SGLT2i therapy was absent in patients with an eGFR <60 mL/min/1.73 m^2^ [133]. 

Uric acid is not transported by SGLT2 [136], and SGLT2is do not interact with transporters involved in renal uric acid reabsorption (i.e., uric acid transporter 1, organic anion transporters 4 and 10, glucose transporter 9, and sodium-coupled monocarboxylate transporter) [136]. Thus, the uric acid-lowering effects of SGLT2is are thought to be mediated by the urinary excretion of uric acid secondary to glucosuria [136]. 

In patients with HFrEF enrolled in EMPEROR-HF, treatment with an SGLT2i was associated with more rapid reductions in serum uric acid levels than those with placebo treatment, which were reflected in a lower incidence of hyperuricemic events (acute gout, gouty arthritis, and initiation of anti-gout therapy) [137]. Moreover, the significant beneficial effects of empagliflozin therapy on the primary composite endpoint were not influenced by baseline serum uric acid levels [137]. 

### 3.10. Effects on Intracellular Sodium

Modulation of intracellular sodium through inhibition of the sodium–hydrogen exchanger (NHE) in both the kidneys and the myocardium may contribute to the cardiovascular and renal benefits of SGLT2i therapy [138,139]. Patients with T2D and HF have increased NHE activity that may contribute to resistance to diuretics and endogenous natriuretic peptides [139]. Reductions in cardiac injury, hypertrophy, fibrosis, remodeling, and systolic dysfunction observed with empagliflozin have been suggested to result from inhibition of the NHE [138]. In support of this hypothesis, empagliflozin has been shown to reduce intracellular sodium and calcium concentrations in isolated rabbit cardiomyocytes; empagliflozin, dapagliflozin, and canagliflozin reduced intracellular sodium in mouse cardiomyocytes, presumably through direct action on the NHE [140,141]; and empagliflozin reduced intracellular sodium in human umbilical vein endothelial cells and human coronary artery endothelial cells [142]. In contrast, empagliflozin and other SGLT2i agents did not inhibit the NHE and had no effect on intracellular sodium concentrations in isolated rat ventricular cardiomyocytes [143]. 

### 3.11. Anti-Inflammatory, Antiplatelet, and Antioxidant Effects

Cardiovascular and renal complications in patients with T2D are significantly driven by chronic low-grade inflammation mediated by the NOD-like receptor pyrin domain-containing protein 3 inflammasome and oxidative stress [144]. SGLT2is may have anti-inflammatory effects that reverse fibrosis, slow CKD progression, and provide cardioprotective effects [145,146]. Serum inflammatory markers influenced by SGLT2is are listed in Table 2 [145,146]. 

SGLT2is reduce inflammation and oxidative stress in vitro and in animal models [147,148,149]. The anti-inflammatory effects may result, in part, from inhibition of the NHE and attenuation of the sodium levels in human endothelial cells [142]. In patients with concomitant T2D and coronary artery disease, treatment with 10 mg/day of empagliflozin for 6 months mitigated inflammation, platelet reactivity, and oxidative stress and was associated with glycemic improvement in the randomized, double-blind, placebo-controlled EMPA-CARD study [150]. Improvements included significant reductions in markers of inflammation (interleukin-6, interleukin-1β, and high-sensitivity C-reactive protein), platelet reactivity (expression of P-selectin), and oxidative burden (reactive oxygen species) and increases in antioxidant levels (superoxide dismutase, glutathione, and total antioxidant capacity). 

Short-term treatment with an SGLT2i significantly decreased platelet reactivity in patients with stable coronary artery disease and T2D who were receiving dual antiplatelet therapy (aspirin plus clopidogrel) in the open-label EFFECT (empagliflozin) [151] and EDGE (dapagliflozin) [152] pilot studies.

### 3.12. Effects on Lipolysis and Ketone bodies

It is hypothesized that alterations in lipolysis and ketone metabolism and the renal handling of ketone bodies may contribute to the cardio- and reno-protective effects of SGLT2is [153]. SGLT2i-induced glycosuria results in a net energy loss, thereby producing a fasting-like metabolic environment. The utilization of glucose decreases, whereas the utilization of free fatty acids (FFA), ketone bodies, and branched chain amino acids (BCAAs) increases. Elevated ketone body levels represent an additional energy source that contributes to improved performance of the heart and kidney. Empagliflozin has been shown to increase circulatory FFA, blood ketones, and BCAAs in patients with T2D [154,155,156]. In addition, experiments in animal models suggest that the SGLT2i-induced shift to a fasting-like metabolic response attenuates the mechanistic target of the rapamycin (mTOR) pathway and improves mitochondrial function [153].

The fasting-like metabolic environment and production of ketone bodies may also contribute to rare cases of euglycemic diabetic ketoacidosis during treatment with SGLT2is. The incidence of this complication was similar or lower in patients treated with an SGLT2i than that in patients receiving a placebo in several cardiovascular and renal outcome trials, including EMPEROR-Reduced [24], EMPEROR-Preserved [22], DAPA-HF [23], and DAPA CKD [27], and higher in patients treated with an SGLT2i than that in those treated with a placebo in other trials, including EMPA-KIDNEY [29], CREDENCE [28], and SCORED [20]. Given the potential seriousness of this condition, clinicians should be aware of the possibility that patients may present with diabetic ketoacidosis during treatment with an SGLT2i [157].

## 4. Conclusions

In patients with and without T2D, SGLT2is confer cardiovascular and renal benefits that result from complex pharmacological mechanisms. These include effects that are independent of their blood glucose-lowering activity. Enhanced tubuloglomerular feedback plays a central role in protecting the kidney and attenuating the decline in GFR and progression of albuminuria. The link between inhibition of SGLT2 in the kidney and reductions in clinical outcomes (hospitalizations, progression of renal disease, mortality) is multifactorial and involves diverse elements, including the inhibition of central SNS outflow and improved hemodynamics. Although initially developed as antihyperglycemic agents, SGLT2is play an increasingly important role in the management of HF and CKD that extends beyond the treatment of T2D.

## Figures and Tables

**Table 1:** The effect of SGLT2i therapy on cardiovascular and renal outcomes in clinical trials.

Study Name	Drug	Population	CV Outcomes, HR (95% CI)	Renal Outcomes, HR (95% CI)
CVOTs in patients with T2D				
CANVAS [15]	Canagliflozin	Age ≥30 y with T2D and established CVD OR Age ≥50 y with T2D and ≥2 CVD risk factors (N = 10,142)	MACE ^a,b^: 0.86 (0.75–0.97) *p* < 0.001 for noninferiority, *p* = 0.02 for superiority; CV death or HHF: 0.78 (0.67–0.91); HHF: 0.67 (0.52–0.87); CV death: 0.87 (0.72–1.06)	Progression of albuminuria: 0.73 (0.67–0.79); 40% reduction in eGFR, RRT initiation, or death from renal causes: 0.60 (0.47–0.77)
DECLARE–TIMI 58 [18]	Dapagliflozin	Age ≥40 y with T2D and established CVD OR Age ≥55 y (men) or ≥60 y (women) with T2D and ≥1 CVD risk factor (N = 17,160)	MACE ^a,b^: 0.93 (0.84–1.03) *p* < 0.001 for noninferiority, *p* = 0.17 for superiority; CV death or HHF ^a^: 0.83 (0.73–0.95) *p* = 0.005 for superiority; HHF: 0.73 (0.61–0.88); CV death: 0.98 (0.82–1.17)	≥40% reduction in eGFR to <60 mL/min/1.73 m^2^, ESKD, or death from CV or renal causes: 0.76 (0.67–0.87) ≥40% reduction in eGFR to <60 mL/min/1.73 m^2^, ESKD, or death from renal causes: 0.53 (0.43–0.66)
EMPA-REG OUTCOME [14,17]	Empagliflozin	Age ≥18 y with T2D and established CVD (N = 7020)	MACE ^a,b^: 0.86 (0.74–0.99) *p* < 0.001 for noninferiority, *p* = 0.04 for superiority; MACE ^b^ or hospitalization for UA: 0.89 (0.78–1.01) *p* < 0.001 for noninferiority, *p* = 0.08 for superiority; CV death or HHF: 0.66 (0.55–0.79) *p* < 0.001 HHF: 0.65 (0.50–0.85) *p* = 0.002; CV death: 0.62 (0.49–0.77) *p* < 0.001	Incident or worsening nephropathy^c^ or CV death: 0.61 (0.55–0.69) *p* < 0.001; Incident or worsening nephropathy^c^: 0.61 (0.53–0.70) *p* < 0.001; Doubling of sCr with eGFR ≤45 mL/min/1.73 m^2^, RRT initiation, or death from renal causes: 0.54 (0.40–0.75) *p* < 0.001
VERTIS CV [19]	Ertugliflozin	Age ≥40 y with T2D and established CVD (N = 8246)	MACE ^a,b^: 0.97 (0.85–1.11) *p* < 0.001 for noninferiority, CV death or HHF: 0.88 (0.75–1.03) *p* = 0.11; HHF: 0.70 (0.54–0.90); CV death: 0.92 (0.77–1.11)	Doubling of sCr, RRT initiation, or death from renal causes: 0.81 (0.63–1.04)
Renal outcomes trials				
CREDENCE [28]	Canagliflozin	Age ≥30 y with T2D and CKD^d^ (N = 4401)	MACE ^b^: 0.80 (0.67–0.95) *p* = 0.01; CV death or HHF: 0.69 (0.57–0.83) *p* < 0.001; HHF: 0.61 (0.47–0.80) *p* < 0.001; CV death: 0.78 (0.61–1.00) *p* = 0.05	ESKD, doubling of sCr, or death from renal or CV causes ^a^: 0.66 (0.53–0.81) *p* < 0.001; Doubling of sCr: 0.60 (0.48–0.76) *p* < 0.001; ESKD: 0.68 (0.54–0.86) *p* = 0.002
DAPA-CKD [27]	Dapagliflozin	Age ≥18 y with CKD^e^ with or without T2D (N = 4094) Patients without T2D: 32.5% of total population	CV death or HHF: 0.71 (0.55–0.92) *p* = 0.009; CV death: 0.81 (0.58–1.12); All-cause mortality: 0.69 (0.53–0.88) *p =* 0.004	Sustained ≥50% reduction in eGFR, ESKD, or death from renal or CV causes ^a^: 0.61 (0.51–0.72) *p* < 0.001; Sustained ≥50% reduction in eGFR, ESKD, or death from renal causes: 0.56 (0.45–0.68) *p* < 0.001; ≥50% reduction in eGFR: 0.53 (0.42–0.67); ESKD: 0.64 (0.50–0.82)
EMPA-KIDNEY [29]	Empagliflozin	Adults with CKD^f^ with or without T2D (N = 6609) Patients without T2D: 54% of total population	HHF or death from CV causes: 0.84 (0.67–1.07) *p* = 0.15; All-cause mortality: 0.87 (0.70–1.08) *p* = 0.21; All-cause hospitalization 0.86 (0.78–0.95) *p =* 0.003; Death from CV causes: 0.84 (0.60–1.19)	Kidney disease progression or death from CV causes: 0.72 (0.64–0.82) *p* < 0.001; Kidney disease progression: 0.71 (0.62–0.81); ESKD or death from CV causes: 0.73 (0.59–0.89)
SCORED [20]	Sotagliflozin	Age ≥18 y with T2D, CKD^g^, and additional CV risk factors^h^ (N = 10,584)	CV death, HHF, or urgent HF visit ^a^: 0.74 (0.63–0.88) *p* < 0.001 HHF or urgent HF visit: 0.67 (0.55–0.82) *p* < 0.001; CV death: 0.90 (0.73–1.12) *p* = 0.35; MACE: 0.77 (0.65–0.91)	Sustained ≥50% reduction in eGFR for ≥30 d, long-term dialysis, renal transplantation, or sustained eGFR of <15 mL/min/1.73 m^2^ for ≥30 d: 0.71 (0.46–1.08)
HF outcome trials				
DAPA-HF [23]	Dapagliflozin	Age ≥18 y with NYHA class II–IV HFrEF (EF ≤40%) (N = 4744) Patients without T2D (including previously undiagnosed): *n* = 2605 (54.9% of total population)	Worsening HF^h^ or CV death^a^: 0.74 (0.65–0.85) *p* < 0.001; CV death or HHF: 0.75 (0.65–0.85) *p* < 0.001; Worsening HF^i^: 0.70 (0.59–0.83); HHF: 0.70 (0.59–0.83); Urgent HF visit: 0.43 (0.20–0.90); CV death: 0.82 (0.69–0.98); ΔKCCQ: 1.18 (1.11–1.26) *p* < 0.001; All-cause mortality: 0.83 (0.71–0.97)	Worsening renal function ^j^: 0.71 (0.44–1.16)
DELIVER [25,30]	Dapagliflozin	Age ≥40 y with NYHA class II–IV HFpEF (EF > 40%) with or without T2D (N = 6263) Patients without T2D: *n* = 3457 (55.2% of total population)	CV death, HHF or urgent HF visit^a^: 0.82 (0.73–0.92) *p* < 0.001; HF events (HHF/urgent HF visit): 0.79 (0.69–0.91); CV death: 0.88 (0.74–1.05); ΔKCCQ: 1.11 (1.03–1.21) *p* = 0.009	Mean difference (95% CI) in eGFR slope change per year vs. placebo (from baseline to end of trial): 0.5 mL/min/1.73 m^2^ (0.1–0.9) *p* = 0.01; Mean difference (95% CI) in eGFR slope change per year vs. placebo (from month 1 to end of trial): 1.4 mL/min/1.73 m^2^ (1.0–1.8) *p* = 0.001; Composite renal outcome ^k^: 1.08 (0.79–1.49)
EMPEROR-Reduced [24]	Empagliflozin	Age ≥18 y with NYHA class II–IV HFrEF (EF ≤ 40%) (N = 3730) Patients without T2D: *n* = 1874 (50.2% of total population)	CV death or HHF ^a^: 0.75 (0.65–0.86) *p* < 0.001; HHF: 0.69 (0.59–0.81); CV death: 0.92 (0.75–1.12)	Composite renal outcome^l^: 0.50 (0.32–0.77)
EMPEROR-Preserved [22]	Empagliflozin	Age ≥18 y with NYHA class II–IV HFpEF (EF > 40%) (N = 5988) Patients without T2D: *n* = 3050 (50.9% of total population)	CV death or HHF ^a^: 0.79 (0.69–0.90) *p* < 0.001; HHF: 0.71 (0.60–0.83); CV death: 0.91 (0.76–1.09)	Mean difference (95% CI) in eGFR slope change per year vs. placebo: 1.36 mL/min/1.73 m^2^ (1.06–1.66) *p* < 0.001; Composite renal outcome^j^: 0.95 (0.73–1.24)
SOLOIST-WHF [21]	Sotagliflozin	Age 18–85 y with T2D and hospitalized for signs and symptoms of HF, treated with intravenous diuretics (N = 1222)	CV death, HHF, or urgent HF visit^a^: 0.67 (0.52–0.85) *p* < 0.001; HHF or urgent HF visit: 0.64 (0.49–0.83) *p* < 0.001; CV death: 0.84 (0.58–1.22) *p* = 0.36	Difference (95% CI) in LS mean change in eGFR: –0.16 (–1.30, +0.98) mL/min/1.73 m^2^

^a^ Primary composite outcome (other outcomes are secondary). ^b^ Defined as the composite outcome of CV death, non-fatal myocardial infarction, or non-fatal stroke. ^c^ Defined as the composite outcome of progression to macroalbuminuria (UACR > 300 mg/g), doubling of sCr accompanied by an eGFR of ≤45 mL/min/1.73 m^2^, initiation of RRT, or death from renal causes. ^d^ Defined as an eGFR of between 30–90 mL/min/1.73 m^2^ and a UACR of between 300–5000 mg/g. ^e^ Defined as an eGFR of between 25–75 mL/min/1.73 m^2^ and a UACR of between 200–5000 mg/g. ^f^Defined as an eGFR of between 20–45 mL/min/1.73 m^2^ irrespective of albuminuria or between 45–90 mL/min/1.73 m^2^ and a UACR ≥200 mg/g. ^g^Defined as an eGFR of between 25–60 mL/min/1.73 m^2^. ^h^≥1 major CV risk factor in participants aged ≥18 y or ≥2 minor CV risk factors in participants aged ≥55 y. ^i^ Defined as hospitalization or an urgent visit resulting in intravenous therapy for HF. ^j^ Defined as the composite outcome of ≥50% reduction in eGFR sustained for ≥28 days, ESKD, or death from renal causes. ^k^ Defined as the composite outcome of ≥50% reduction in eGFR, ESKD, or death from renal causes. ^l^Defined as chronic dialysis or renal transplantation, a sustained reduction of ≥40% in eGFR, or a sustained eGFR of <15 mL/min/1.73 m^2^ (baseline eGFR of ≥30 mL/min/1.73 m^2^) or <10 mL/min/1.73 m^2^ (baseline eGFR of <30 mL/min/1.73 m^2^). CI, confidence interval; CKD, chronic kidney disease; CV, cardiovascular; CVD, cardiovascular disease; CVOT, cardiovascular outcomes trial; d, days; eGFR, estimated glomerular filtration rate; EF, ejection fraction; ESKD, end-stage kidney disease; HF, heart failure; HFpEF, heart failure with preserved ejection fraction; HFrEF, heart failure with reduced ejection fraction; HHF, hospitalization for heart failure; HR, hazard ratio; ΔKCCQ, change in Kansas City Cardiomyopathy Questionnaire score at 8 months; LS, least-squares; MACE, major adverse cardiovascular event; NYHA, New York Heart Association; RRT, renal replacement therapy; sCr, serum creatinine; SGLT2i, sodium-glucose cotransporter-2 inhibitor; T2D, type 2 diabetes; UA, unstable angina; UACR, urinary albumin-to-creatinine ratio; y, year.

**Table 2:** Changes in inflammation and fibrosis markers in patients with T2D treated with SGLT2is [145,146]. Increase in level is marked as “↑”, decrease as “↓”, and significant changes (*p* < 0.05) as “*”.

Inflammatory Marker	Canagliflozin	Dapagliflozin	Empagliflozin
Adiponectin ^a^	↑ *	↑ *	
Leptin	↓	↓	
hsCRP	↓	↓ *	↓
TNF-α	↓	↓ *	↓ *
TNFR1	↓ *		
IL-6	↓ *		↓ *
IFN-γ			↓ *
MMP7	↓ *		
FN1	↓ *		
CCL5	No change		
HGF	↓		
VEGFA	↓300 mg (but not 100 mg)		
FABP1	↓		

^a^ Only adiponectin levels were increased with SGLT2is. CCL, C-C motif chemokine ligand, FABP1, fatty acid binding protein 1; FN1, fibronectin 1; HGF, hepatocyte growth factor; hsCRP, high-sensitivity C-reactive protein; IFN-γ: interferon-gamma; IL-6, interleukin-6; MMP, matrix metalloproteinase; SGLT2i, sodium-glucose cotransporter 2 inhibitor; T2D, type 2 diabetes; TNF-α, tumor necrosis factor-alpha; TNFR1, TNF receptor 1; VEGFA, vascular endothelial growth factor A.

## Data Availability

Not applicable.
